# The Post-thrombotic Syndrome-Prevention and Treatment: VAS-European Independent Foundation in Angiology/Vascular Medicine Position Paper

**DOI:** 10.3389/fcvm.2022.762443

**Published:** 2022-02-24

**Authors:** Benilde Cosmi, Agata Stanek, Matja Kozak, Paul W. Wennberg, Raghu Kolluri, Marc Righini, Pavel Poredos, Michael Lichtenberg, Mariella Catalano, Sergio De Marchi, Katalin Farkas, Paolo Gresele, Peter Klein-Wegel, Gianfranco Lessiani, Peter Marschang, Zsolt Pecsvarady, Manlio Prior, Attila Puskas, Andrzej Szuba

**Affiliations:** ^1^Division of Angiology and Blood Coagulation, Department of Specialty, Diagnostic and Experimental Medicine, S. Orsola Malpighi University Hospital Research Institute IRCSS, University of Bologna, Bologna, Italy; ^2^Inter-University Research Center on Vascular Diseases & Angiology Unit, University of Milan, L Sacco Hospital, VAS-European Independent Foundation in Angiology/Vascular Medicine, Milan, Italy; ^3^Department of Internal Medicine, Angiology and Physical Medicine, Faculty of Medical Sciences in Zabrze, Medical University of Silesia, Bytom, Poland; ^4^Department for Vascular Diseases, Medical Faculty of Ljubljana, University Medical Center Ljubljana, Ljubljana, Slovenia; ^5^Department of Cardiovascular Medicine, Gonda Vascular Center, Mayo Clinic, Rochester, MN, United States; ^6^Cardiovascular Medicine, OhioHealth/Riverside Methodist Hospital, Columbus, OH, United States; ^7^Division of Angiology and Hemostasis, Geneva University Hospitals and Faculty of Medicine, Geneva, Switzerland; ^8^Department for Vascular Disease, University Medical Center Ljubljana, Ljubljana, Slovenia; ^9^Michael Lichtenberg, Klinikum Hochsauerland, Arnsberg, Germany; ^10^Unit of Angiology, Department of Medicine - University of Verona, Cardiovascular and Thoracic Department, Verona University Hospital, Verona, Italy; ^11^Department of Angiology, Szent Imre University Teaching Hospital, Budapest, Hungary; ^12^Department of Medicine and Surgery, University of Perugia, Perugia, Italy; ^13^Angiologic Clinic, Interdisciplinary Center of Vascular Medicine, Klinikum Ernst von Bergmann, Potsdam, Germany; ^14^Angiology Unit, Department of Internal Medicine, Città Sant'Angelo Hospital, Pescara, Italy; ^15^Department of Internal Medicine, Central Hospital of Bolzano (SABES-ASDAA), Bolzano, Italy; ^16^2nd Department of Internal Medicine - Vascular Center, Flor Ferenc Teaching Hospital, Kistarcsa, Hungary; ^17^Angio Center-Vascular Medicine Private Clinic, Tirgu Mures, Romania; ^18^Department of Angiology, Hypertension and Diabetology, Wroclaw Medical University, Wroclaw, Poland

**Keywords:** post-thrombotic syndrome, post-phlebitic syndrome, deep vein thrombosis, prevention, diagnosis, treatment

## Abstract

**Importance:**

The post-thrombotic syndrome (PTS) is the most common long-term complication of deep vein thrombosis (DVT), occurring in up to 40–50% of cases. There are limited evidence-based approaches for PTS clinical management.

**Objective:**

To provide an expert consensus for PTS diagnosis, prevention, and treatment.

**Evidence-Review:**

MEDLINE, Cochrane Database review, and GOOGLE SCHOLAR were searched with the terms “post-thrombotic syndrome” and “post-phlebitic syndrome” used in titles and abstracts up to September 2020.

**Filters Were:**

English, Controlled Clinical Trial / Systematic Review / Meta-Analysis / Guideline. The relevant literature regarding PTS diagnosis, prevention and treatment was reviewed and summarized by the evidence synthesis team. On the basis of this review, a panel of 15 practicing angiology/vascular medicine specialists assessed the appropriateness of several items regarding PTS management on a Likert-9 point scale, according to the RAND/UCLA method, with a two-round modified Delphi method.

**Findings:**

The panelists rated the following as appropriate for diagnosis: 1-the Villalta scale; 2- pre-existing venous insufficiency evaluation; 3-assessment 3–6 months after diagnosis of iliofemoral or femoro-popliteal DVT, and afterwards periodically, according to a personalized schedule depending on the presence or absence of clinically relevant PTS. The items rated as appropriate for symptom relief and prevention were: 1- graduated compression stockings (GCS) or elastic bandages for symptomatic relief in acute DVT, either iliofemoral, popliteal or calf; 2-thigh-length GCS (30–40 mmHg at the ankle) after ilio-femoral DVT; 3- knee-length GCS (30–40 mmHg at the ankle) after popliteal DVT; 4-GCS for different length of times according to the severity of periodically assessed PTS; 5-catheter-directed thrombolysis, with or without mechanical thrombectomy, in patients with iliofemoral obstruction, severe symptoms, and low risk of bleeding. The items rated as appropriate for treatment were: 1- thigh-length GCS (30–40 mmHg at the ankle) after iliofemoral DVT; 2-compression therapy for ulcer treatment; 3- exercise training. The role of endovascular treatment (angioplasty and/or stenting) was rated as uncertain, but it could be considered for severe PTS only in case of stenosis or occlusion above the inguinal ligament, followed by oral anticoagulation.

**Conclusions and Relevance:**

This position paper can help practicing clinicians in PTS management.

## Introduction

Post-thrombotic syndrome (PTS) is the most common long-term complication of deep vein thrombosis (DVT) occurring in up to 40–50% of patients, primarily due to impaired thrombus resolution with persistent venous outflow obstruction and secondary valvular incompetence (1). PTS has not been routinely considered as an outcome of the large number of randomized clinical trials which have investigated pharmacological strategies for the prevention and treatment of venous thromboembolism (VTE), which includes DVT and PE in the last 30 years (2–4) and only secondary *post-hoc* analyses are available (5). Unfortunately there is very limited evidence regarding a number of issues in PTS management, and what evidence exists is of very low quality.

The limited evidence available and the many areas of uncertainty also imply a wide spectrum of variations and heterogeneity in PTS clinical management across different countries.

This prompted VAS-European Independent Foundation in Angiology/Vascular Medicine to launch a project for a position paper on the appropriateness of interventions for PTS involving practicing expert clinicians from many different countries.

The concept of appropriateness refers to the relative weight of the benefits and harms of a medical or surgical intervention (6). An appropriate procedure is one in which “the expected health benefit (e.g., increased life expectancy, relief of pain, reduction in anxiety, improved functional capacity) exceeds the expected negative consequences (e.g., mortality, morbidity, anxiety, pain, time lost from work) by a sufficiently wide margin that the procedure is worth doing, exclusive of cost” (6).

VAS-European Independent Foundation in Angiology/Vascular Medicine is a non-profit scientific organization (Transparency European Union Register Number: 818165941069-15) which was established in 1991 (as European Working Group). VAS established programs on European education and training (UEMS accreditation), promoting collaborative research as well as awareness in the area of vascular medicine/angiology (www.vas-int.net). To enforce stable collaborations in Europe and internationally, VAS defined stable partnerships with >50 scientific societies organizations and Universities in Europe and at international level. VAS is present in more than 40 countries and it has established networks focused on actions and campaigns, aimed at improving and qualifying competences and attention on vascular disease, as well as suggesting concrete changes on health systems approach to vascular diseases and their prevention for the benefit of patients and populations.

The aim of VAS position paper was to provide practical indications to the busy clinician for diagnosis, prevention, and treatment of PTS after DVT of the lower limbs.

## Methods

We used the RAND/UCLA appropriateness method and a two-round modified Delphi method (6). A multidisciplinary group of expert practicing clinicians were recruited to conduct a literature review (evidence synthesis team) composed of eight practicing angiology/vascular medicine specialists from seven different European Countries and from USA. MEDLINE, Cochrane Database review and GOOGLE SCHOLAR were searched from up to September 2020 with terms used in titles and abstracts: “post-phlebitic syndrome,” “diagnosis,” “prevention,” and “treatment.” Filters were: English, Controlled Clinical Trial / Systematic Review / Meta-Analysis / Guideline. Only prospective clinical trials examining PTS diagnosis, and randomized clinical trials and systematic reviews that examined prevention and treatment of PTS, published in the English language, were included. Abstracts, conference proceedings, review paper, observational or retrospective cohort studies for prevention or treatment, editorials and commentaries were excluded.

Following the search, duplicates were removed. Titles and abstracts were screened for assessment against review inclusion criteria. Full text of selected citations was assessed in detail against the inclusion criteria and, out of 496 citations, three prospective studies for diagnosis, five systematic reviews, and four randomized clinical trials not included in the systematic reviews were selected. Any disagreements that arose between the reviewers were resolved through discussion.

Methodological assessment was conducted according to the American College of Cardiology Foundation/ American Heart Association Task Force on Practice Guidelines (7) and The Rational Clinical Examination (8). Methodological assessment was completed for systematic reviews and primary studies not included in the systematic reviews. In fact primary studies included in the systematic reviews had been assessed for risk of bias when included in the original systematic reviews. Appendix 1 reports the results of this review.

Based on this review, a Likert 9-point scale was constructed for each of 29 items regarding PTS diagnosis, prevention and treatment. Forms were sent to a 15 member expert panel *via* e-mail. The expert panel was composed of 13 angiology/vascular medicine specialists and two internal medicine specialists from seven different European countries, some of them also members of the VAS Advisory board with a large clinical experience in qualified centers.

Each panelist rated each item separately and e-mailed the rated items to the moderator (BC) (first round). Items were classified into three levels of appropriateness (Table 1).

**Table 1:** Ratings of proposed items with medians and disagreement.

**PTS diagnosis and surveillance**	**Median**	**Disagreement**
1- The Villalta scale is recommended for diagnosis and severity classification of PTS	7	No
2- The Ginsberg scale is recommended for diagnosis and severity classification of PTS	5	Yes
3- The Brandjes scale is recommended for diagnosis and severity classification of PTS	5	No
4- The CEAP scale is recommended for diagnosis and severity classification of PTS	5	No
5- Preexisting venous insufficiency (e.g., contralateral limb) should be taken into account for classifying PTS severity after DVT	7	No
6- PTS should be assessed 1 month after the diagnosis of iliofemoral DVT	4	Yes
7- PTS should be assessed 1 month after the diagnosis of popliteal or calf DVT	4	Yes
8- PTS should be assessed 6 months after the diagnosis of iliofemoral DVT	8	No
9- PTS should be assessed 6 months after the diagnosis of popliteal or calf DVT	7	No
10- PTS should be assessed periodically (e.g., 6 months) and for at least 2 years since the diagnosis of proximal or calf DVT	7	No
**PTS symptom mangement and prevention**	**Median**	**Disagreement**
1- Graduated compression stockings (GCS) or elastic bandages are recommended for symptomatic relief in acute DVT	8	No
2- Knee length GCS (40 mmHg at the ankle) are recommended after iliofemoral DVT	6	No
3- Thigh-length GCS (40 mmHg at the ankle) are recommended after iliofemoral DVT	7	No
4- Knee length GCS (40 mmHg at the ankle) are recommended after popliteal or calf DVT	7	No
5- Thigh length GCS (40 mmHg at the ankle) are recommended after popliteal or calf DVT	4	No
6- GCS are recommended for different lengths of time according to the severity of periodically assessed PTS	7	No
7- Catheter-directed thrombolysis, with or without mechanical thrombectomy, are appropriate in patients with iliofemoral obstruction, severe symptoms, and a low risk of bleeding	7	No
8- Catheter-directed thrombolysis, with or without mechanical thrombectomy, are appropriate in patients with popliteal obstruction, severe symptoms, and a low risk of bleeding	4	No
**PTS Treatment**	**Median**	**Disagreement**
1- Thigh length GCS (30–40 mmHg at the ankle) are recommended after iliofemoral DVT	7	No
2- Knee length GCS (30–40 mmHg at the ankle) are recommended after iliofemoral DVT	6	No
3- Thigh-length GCS (30–40 mmHg at the ankle) are recommended after popliteal or calf DVT	3	No
4- Knee length GCS (30–40 mmHg at the ankle) are recommended after popliteal or calf DVT	7	No
5- Compression therapy is recommended for ulcer treatment	9	No
6- Exercise training is recommended for PTS treatment	7	No
7- Endovascular treatment (angioplasty and/or stenting) is recommended for the treatment of severe PTS	6	No
8- Oral anticoagulation is recommended after endovascular treatment with stenting	7	No
9- Long term oral anticoagulation is recommended after endovascular treatment with stenting	6	No
10- Open surgical reconstruction and hybrid operations are appropriate for the treatment of severe PTS	4	No
11- Veno-active drugs are recommended	6	No

*Appropriate: panel median of 7–9, without disagreement on the final appropriateness scale: it would be considered improper care not to provide this service, and there is a reasonable chance that this procedure will benefit the patient. The benefit to the patient is not small*.

*Uncertain: panel median of 4–6 OR any median with disagreement; Inappropriate: panel median of 1–3, without disagreement*.

Indications were classified into three levels of appropriateness, using the following definitions (6):

- *Appropriate*: panel median of 7–9, without disagreement on the final appropriateness scale. It would be considered improper care not to provide this service, and there is a reasonable chance that this procedure will benefit the patient (A procedure could be appropriate if it had a low likelihood of benefit but few risks; such procedures would not be necessary). The benefit to the patient is not small (A procedure could be appropriate if it had a minor but almost certain benefit, but it would not be necessary).
- *Uncertain*: panel median of 4–6 OR any median with disagreement
- *Inappropriate*: panel median of 1–3, without disagreement

And the agreement of all ratings was calculated with the Interpercentile Range Adjusted for Symmetry (IPRAS) (6). If the Interpercentile Range of a particular indication is larger than the IPRAS of that particular indication, it is rated with disagreement. This method allows for any number of participant responses and better accounts for dispersion and higher weights on the extremes than traditional methods (9).

The second round involved a face-to-face web-based virtual meeting of panelists with the moderator to debate the median ratings and disagreements from all panelists and to propose items for the final statements.

## Results

Table 1 shows ratings and disagreements. During the second round, of the 26 items for which there was agreement, 15 were accepted with no change, two were modified and retained, and nine were deleted. The three items for which there was disagreement were deleted. The final total was 17 items.

### VAS Position Statements

#### PTS Diagnosis and Surveillance

1- The Villata Scale (VS) is appropriate for the diagnosis and classification of PTS severity.
2- It is appropriate to assess pre-existing venous insufficiency (e.g., contralateral limb) for classifying PTS severity after DVT.
3- It is appropriate to assess PTS at least 3–6 months after the diagnosis of iliofemoral or femoro-popliteal DVT, and afterwards according to a personalized schedule depending on the presence or absence of clinically relevant PTS at these time-points.

There is no specific recommended time limit to diagnose PTS and studies have followed up patients for two 2 years or longer. Initial symptoms and signs of the acute phase may require sometime to subside. As a result, the diagnosis of PTS should be deferred until 3–6 months. Afterwards, the timing of surveillance is also related to the severity of PTS at these time points, also considering risk factors for PTS and patients' characteristics (DVT initial extension, BMI, life style).

#### PTS Symptom Management

1- Graduated compression stockings (GCS) or elastic bandages are appropriate for symptomatic relief in acute DVT, either iliofemoral, popliteal, or calf.

Compression can ameliorate limb pain and swelling in both proximal and calf DVT. Ideally, elastic bandages are more appropriate in severely swollen limbs in the first few days, although not always feasible. After reducing the swelling with compression bandages, GCS can be applied. The size of compression stockings should be taken on the limb contralateral to the DVT to avoid stockings becoming too large after oedema has subsided.

2- Catheter-directed thrombolysis, with or without mechanical thrombectomy, is appropriate in patients with iliofemoral obstruction, severe symptoms, and a low risk of bleeding.

It is appropriate, especially in young subjects with iliofemoral DVT, for symptomatic relief (especially in Iliac Vein Compression syndrome) and improvement of quality of life.

The CaVent study on CDT had a long follow-up and reported a significant reduction in PTS in subjects with iliofemoral DVT treated with CDT albeit with an increased risk of bleeding.

#### PTS Prevention

1- Thigh-length GCS (30–40 mmHg at the ankle) are appropriate for the prevention of PTS after iliofemoral DVT.

Studies on the use of GCS for PTS prevention have produced conflicting results and a Cochrane meta-analysis concluded that the use of GCS led to a clinically significant, although non statistically significant, reduction in the incidence of PTS albeit with no reduction in the incidence of severe PTS and no clear difference in DVT recurrence or PE.

Studies have evaluated knee-high GCS to prevent PTS, as they are more comfortable and easy to wear. Thigh-high GCS are also available, although less comfortable, and they may be employed during the initial 6–12 months, while knee-high GCS could be employed afterward. A personalized choice of stockings could be considered according to DVT extension and development of PTS.

2- Knee-length GCS (30–40 mmHg at the ankle) are appropriate for the prevention of PTS after popliteal DVT.

Knee-length stockings are more comfortable, and they increase patient compliance with GCS.

The correct information and continuous guidance of the patient is paramount to increase compliance. A reduction of the degree of compression may be considered to improve compliance. Calf DVT does not deserve stockings for prevention, but only for symptom relief.

3- GCS are appropriate for different lengths of time according to the severity of periodically assessed PTS.

The duration of compression stockings should be individualized according to the severity of PTS as assessed over time. Thigh-high GCS could be used in certain patients, such as those with extensive iliofemoral DVT, skin induration, or secondary lymphedema (“phlebolymphedema”).

#### PTS Treatment

1- Thigh length GCS (30–40 mmHg at the ankle) are appropriate for PTS treatment after iliofemoral DVT.
2- Knee length GCS (30–40 mmHg at the ankle) are appropriate for PTS treatment after popliteal DVT.
3- Compression therapy is appropriate for ulcer treatment.

Treatment of ulcers due to PTS with different types of bandages/stockings is a broader topic, also applying to ulcers due to chronic venous insufficiency, deserving to be addressed separately.

4- Exercise training is appropriate for PTS treatment. Exercise training such as walking is addressed in very few studies, as well as lifestyle changes such as weight loss in overweight or obese subjects.
5- The role of endovascular treatment (angioplasty and/or stenting above the inguinal ligament) is uncertain for the treatment of severe PTS. Such an approach can be considered only in stenosis or occlusion, without severe valve incompetence, and only above the inguinal ligament.
6- Oral anticoagulation is appropriate after endovascular treatment with stenting for PTS treatment.

However, the type and optimal length of anticoagulation is uncertain and still a debated issue.

## Discussion

PTS is the most common complication of DVT; however, many uncertainties remain regarding its diagnosis, prevention, and treatment. This consensus paper provides a framework for the busy clinician, addressing several practical issues of PTS management.

The methodological assessment of the existing studies on PTS rated most of them of low or very low quality. As a result, many issues regarding PTS management deserve further investigation.

PTS diagnosis itself needs further research efforts as more recent studies raise concerns with VS scale, although externally validated and endorsed by scientific societies. Limitations include the subjective measures of its components, the presence of all items in patients with chronic venous insufficiency (CVI) due to primary valvular reflux or secondary superficial venous insufficiency unrelated to DVT (9) and the lack of evaluation of ulcer severity. Since the prevalence of primary CVI is higher in the general population, a significant proportion of DVT patients have pre-existing primary CVI. However, CVI cannot be correctly evaluated at the time of DVT diagnosis although the examination of the contralateral limb could help. The presence of pre-existing VCI could worsen PTS signs and symptoms and lead to overestimation of the severity of PTS during follow-up.

VS has the potential to misclassify or overestimate pre-existing venous disease as PTS (10). However, there is no formal method to account for pre-existing venous insufficiency in the VS. In another study, the authors concluded that common patient complaints and the impact of PTS are not well-reflected in the VS (11). Most recently, Ning et al. noted that VS misclassified those with primary CVI and a history of DVT as having PTS by 42.3% (12). The VS plus revised CEAP could be investigated to incorporate previous venous insufficiency (13).

The timing of PTS surveillance is also not well-defined. Studies have usually evaluated PTS every 6 months after diagnosis for 2 years. Assessment at 1 month after diagnosis is too early, as there might still be symptoms of the acute phase, although one study showed that persisting symptoms after 1 month are associated with a higher risk of PTS. Another option is the SOX-PTS scale combining the VS with BMI plus anatomical extension of PTS at the time of DVT diagnosis which was developed and externally validated to predict PTS occurrence at the time of DVT diagnosis (14). This scale could help identify those subjects who may need more strict surveillance to detect PTS development. Whole leg color Duplex scan ultrasound may be performed at each time point of follow-up (in lying and standing position with standardized provocative maneuvers) but only the basis of worsening symptoms, also to exclude recurrent DVT. Still, it may not reflect the clinical stage of PTS, which requires a physical examination. A Choose Wisely statement of the Society of Vascular Medicine partnered with American Board of Internal Medicine came out with—do not repeat DUS unless there are changes in clinical symptoms (15). Moreover, ultrasound modalities to assess recanalization (e.g., presence of residual thrombosis) and venous reflux are not incorporated in formal scales and not standardized. Methods to standardize the ultrasound results should also require further investigation.

Surveillance with telemedicine to avoid office visits with the self-assessment of VS and quality of life assessment could also be further explored. PTS prevention is still a major debated issue, especially in case of extensive DVT when the interventional approach can be considered. The CaVent study had a long follow-up and reported a significant reduction in PTS in subjects with iliofemoral DVT treated with CDT albeit with an increased risk of bleeding (16).

However, only a minority of the overall population presenting with iliofemoral DVT was enrolled into interventional studies for PTS prevention with pharmacomechanical thrombectomy (PMT) adjunctive to standard anticoagulant treatment vs. standard anticoagulant treatment alone such as in the ATTRACT trial (17), as <2.5% of the screened population were enrolled. Suboptimal technical success rates were observed and this could explain poor outcomes in the intervention arms. However, in these multicentre studies, the technical outcomes could be interpreted as more reflective of routine practice than selected centers of excellence. Several limitations of the ATTRACT study should also be considered, such as a significant heterogeneity of devices and methods employed for clot removal, no clear indication for stenting, the use of only arterial stents were, a long interval between symptom onset and onset of therapy.

In conclusion, the results of the interventional randomized clinical trials such as ATTRACT and CAVA (17, 18) indicate that at the moment, PMT cannot be recommended routinely for DVT of the lower limbs, for which anticoagulation remains the standard treatment. However, the role of interventional therapy for PTS has evolving evidence. The relationship between the technical success of early thrombus removal (and persistent deep venous patency) and clinical outcomes deserves further investigation.

The role of DOACs for PTS prevention is emerging in recent studies and in *post-hoc* analyses evaluating PTS in trials of DVT treatment with DOAC vs. VKA (19). Despite several limitations, in studies on DOACs more than 60–70% of patients were free of PTS and severe manifestations such as skin ulcer and/or other severe and, by definition, intractable manifestations, were observed only in a minority (<5–6%) of subjects after an average of 30-month follow-up in both DOAC and VKA treated patients. These PTS rates at long-term follow-up are similar to those of studies in which thrombolytic therapies were used, such as in the ATTRACT study and are possibly related to a less variable anticoagulant activity in the acute phase of DVT, thus favoring vein recanalization (20). There are no randomized trials comparing interventional approaches to conservative approaches for PTS treatment, and only observational studies are available on the use of venous stents, which therefore cannot be recommended routinely.

In addition, the optimal antithrombotic treatment after venous stenting is still not clearly defined and varies among different studies. As a result, firm indications cannot be extrapolated from such studies. General recommendations include anticoagulant therapy during the intervention and continued after the intervention, usually for 3–6 months. DOACs are being used to an increasing extent, but there is a lack of sufficient experience with these agents (20).

Lifestyle changes such as exercise and BMI control in case of overweight or obesity should also be considered, although very limited evidence exists on these approaches for both PTS prevention and treatment.

## Conclusion

PTS is the most common long term complication of DVT, regular surveillance and conservative medical approach with standard anticoagulation and graduated compression stockings are indicated for PTS prevention in the majority of subjects. Interventional and endovascular approaches for prevention and treatment have limited evidence and should be considered in selected subjects. The statements of this position paper merely reflect the consensus opinion of experts based on low quality evidence in most cases.

## Author Contributions

BC and MC: conception and design, construction of Likert 9-point scale items. BC, AS, MK, PW, RK, MR, PP, ML, and MC: literature search, review with methodological assessments, and evidence synthesis. BC, MC, SD, KF, PG, PW, MK, GL, PM, ZP, PP, MP, AP, MR, ASt, and ASz: rating of items and participation to the two-round modified Delphi consensus. BC and ASt: drafting the article. All authors: critical revision of the article for important intellectual content and final approval. All authors contributed to the article and approved the submitted version.

## Conflict of Interest

BC declares speakers's fees from Instrumentation Laboratory, Werfen IL, Sanofi, Aspen, Bristol-Myers-Squibb, advisory board fees for Viatris, Techdow Farma Italy. ASt declares receiving grants from Metrum Cryoflex, honorario for lectures from Alfa Sigma, President of the Polish Society of Cryotherapy, unpaid. GL reports Leadership or fiduciary role in other board, society, committee or advocacy group, paid or unpaid: Vice President of Italian Society of Angiology and Vascular Disease (2018–2021); consulting fees from Alfa Sigma. PM declares Payment for a lecture from Bayer (2018), President of the Austrian Society of Vascular Medicine (ÖGIA) 2018–2019, unpaid. MP declares payment or honoraria for lectures of Alfa Sigma, Aspen, BMS Pfizer, support for travel from Alfa Sigma. MC Honorary President UEMS, Division of Angiology unpaid. PG is President of the Italian Society for the Study of Haemostasis and Thrombosis (SISET: 2020–2022) unpaid. The remaining authors declare that the research was conducted in the absence of any commercial or financial relationships that could be construed as a potential conflict of interest.

## Publisher's Note

All claims expressed in this article are solely those of the authors and do not necessarily represent those of their affiliated organizations, or those of the publisher, the editors and the reviewers. Any product that may be evaluated in this article, or claim that may be made by its manufacturer, is not guaranteed or endorsed by the publisher.
