# Supplementary material for: The Post-thrombotic Syndrome-Prevention and Treatment: VAS-European Independent Foundation in Angiology/Vascular Medicine Position Paper
Source: Front Cardiovasc Med. 2022 Feb 24;9:762443. doi: 10.3389/fcvm.2022.762443 (PMC8907532; doi:10.3389/fcvm.2022.762443)
Supplement: Supplementary file 1 [file Data_Sheet_1.pdf]

APPENDIX 1: EXPANDED BACKGROUND AND EVIDENCE SYNTHESIS

1. Definition and diagnosis of PTS of the lower limbs

PTS is defined as the presence of new signs and symptoms of venous incompetence after an episode of DVT. These manifestations may be intermittent or persistent, may worsen with standing or walking and improve with rest and leg elevation, with severity ranging from mild symptoms to severe leg pain, skin discoloration, edema, and ulcers that limit daily activities and work. There is no diagnostic gold standard of PTS and no generally accepted definition either, and therefore only clinical evaluation is feasible. Venous valvular incompetence with or without residual post-thrombotic changes may be demonstrated by duplex ultrasound (DUS). But, in the absence of signs and symptoms of PTS, it does not meet the criteria for PTS diagnosis. Three different diagnostic scales for PTS of the lower limbs have been proposed (e-table 1). In 2009, the Subcommittee on Control of Anticoagulation of the Scientific and Standardization Committee of the International Society of Thrombosis and Haemostasis recommended the VS as the standard to diagnose and grade the severity of PTS in clinical research [1]. The VS has been validated in several studies with good discriminative properties among the different severity groups, with good inter-observer reliability, relative ease of use, and responsiveness to clinical changes [1-3]. e-Table 1 shows the methodological assessment of the different scales for PTS diagnosis.

2. Pathogenesis of PTS of the lower limbs

PTS pathophysiology has been related to both venous outflow obstruction due to residual thrombus with venous scarring and venous reflux due to venous valve damage produced by the inflammatory response to thrombosis [4]. Venous hypertension ensues with ambulatory venous pressure failing to fall significantly during walking or exercise (as it does in the healthy state).

Ambulatory venous hypertension increases capillary pressure, disrupting fluid homeostasis, substrate and gas-exchange [5,6]. Furthermore, there is a progressive endothelial dysfunction with increased expression of adhesion molecules such as VCAM, ICAM, and P-selectin leading to

leucocyte and platelet trapping and leucocyte diapedesis [7]. Normal capillary morphology and the microlymphatic network progressively disintegrates, and capillary density decreases [8]. Capillary loss produces a drop in local tcpO2-levels with severe tissue ischemia [5-7]. Ischemia and progressive inflammation have been shown to activate metalloproteinases, which are important mediators of the degenerative processes involved in venous insufficiency and PTS [9]. These macro and microvascular disturbances with chronic inflammatory response to venous hypertension, lead to the formation of a fibrin cuff around capillaries, tissue hypoxia with leukocyte recruitment and increased vascular permeability, all leading to trophic skin changes from hyperpigmentation to atrophy blanche areas, which represent almost capillary free zones with lipodermatosclerosis, ankle swelling, venous claudication, and skin ulceration [10].

Several risk factors are associated to PTS development. Iliac and femoral vein involvement increases the risk by two-fold when compared to isolated calf DVT [11]. Recurrent ipsilateral DVT increases PTS risk by 4 to 6-fold, presumably by damaging compromised venous valves or aggravating venous outflow obstruction [11]. Persistent venous symptoms/signs at one month after acute DVT increases the risk of subsequent PTS [11]. Some patient characteristics such as age, high body mass index, preexisting venous insufficiency, residual post-thrombotic changes on DUS (e.g., 3–6 months after acute DVT) have demonstrated a modest (1.5–2-fold increase) impact in PTS risk [11]. However, the role of residual vein obstruction (RVO) and/or valvular reflux (detected on DUS) as predictors of PTS is less clear [4]. There is conflicting evidence, with some studies indicating a 1.5 fold increased risk of PTS with RVO alone [12,13] and about two-fold increase when both RVO and valvular reflux are present, while other studies have shown that valvular reflux may be associated with a greater risk than RVO in development of PTS [14,15].

However, incomplete or absent recanalization was associated with a higher incidence of PTS in one study of 100 patients followed-up per 12-36 months, while reflux did not influence PTS development [16].

Some studies also indicated that women are at increased risk for PTS than men, although the recanalization rate of DVT is higher in females than in men [17]. A higher incidence of PTS in females could result from hormonal status or more significant thrombus load.

Markers of inflammation have also been investigated for their role in PTS; however, a meta-analysis could not reach firm conclusions due to the heterogeneity of the studies [18].

The duration of anticoagulant treatment is not associated with the risk of developing PTS. However, insufficient anticoagulation treatment with vitamin K antagonists (VKA), as expressed by a sub-therapeutic International Normalized Ratio (INR) of <2.0 for more than 20% of the time in the first three months of treatment, is associated with a two-fold increased risk of PTS (OR, 1.84; 95% CI, 1.13–3.01) [19]. In another study, inadequate anticoagulation with INR in the subtherapeutic range for more than 50% of the time resulted in a 2.7 fold higher risk of developing PTS [20]. The quality of anticoagulation beyond the first three months was shown not to affect PTS development, although it can prevent recurrence and thus the increased risk for PTS [21].

These observations suggest that the initial period of anticoagulation is crucial for reducing PTS development, especially in the first four weeks when the control of anticoagulation with VKA can be suboptimal [22]. This is supported by the results of a randomized trial of continued therapeutic dose low-molecular-weight heparin (LMWH) for DVT treatment compared with warfarin after an initial 5-day course of heparin. In this trial, patients had a 23% reduction in symptoms and signs of PTS at 12 weeks [23]. A meta-analysis of studies of patients continued on therapeutic dose LMWH, as compared with a VKA, confirms a lower incidence of venous ulceration in LMWH-treated patients with improved vein recanalization [24]. This effect may be attributed to the anti-inflammatory properties of LMWH. It is also reasonable to assume that the first four weeks of anticoagulation are crucial for vein recanalization as continued thrombin generation in this time frame may retard clot lysis with resulting connective tissue growth and persistent fibrotic occlusion and venous damage [10].

Adequate and sustained therapeutic anticoagulation in the acute phase of DVT treatment is crucial for achieving adequate clot lysis and reduction of fibrotic thrombus transformation. Direct oral anticoagulants (DOAC) may have a potential advantage over VKA for PTS risk reduction. They might be associated with a sustained and less variable anticoagulant activity, thus favoring vein recanalization. An observational study of 100 patients treated with rivaroxaban had a lower prevalence of PTS than those treated with warfarin (25 % vs. 49 %) [25].

A post-hoc analysis of the Einstein DVT trial showed a non-statistically significant difference in the cumulative PTS incidence rates at 60 months of follow-up between the rivaroxaban group (29%) and the enoxaparin/VKA group (40%) [26]. Similar results have recently been reported in the Danish nationwide registry, including DVT patients treated with rivaroxaban or VKA [27]. More recently, a prospective cohort study compared patients with proximal DVT treated with DOACs to a historical cohort of patients treated with conventional anticoagulation. After propensity score matching, the Odds Ratio (OR) for developing PTS at three years in the DOAC cohort was 0.46 (95% CI, 0.33 to 0.63) compared to the VKA-treated cohort. The rate of PTS was much higher in patients with RVO than in those without, with an advantage for those treated with DOACs compared to VKA [28]. This effect may be attributed to the potentially more stable anticoagulant DOAC effect when compared to VKAs [29].

3. Epidemiology of PTS

PTS epidemiology reflects VTE, which is estimated to affect 104–183 subjects per 100 000 person-years among Caucasians [30], with DVT ranging from 29 to 78 and 45 to 117, per 100 000 person-years, respectively [31,32]. PTS can occur in 40-50% of subjects after DVT, with severe manifestations in 10% and the development of leg ulcers in 1-3% of patients [33]. Leg ulcers tend to recur, and they pose a significant morbidity and health care burden [33].

4. Prevention of PTS

DVT prevention is an appropriate step for preventing PTS. After DVT occurrence, adequate quality of anticoagulant treatment and prevention of recurrence, especially of ipsilateral DVT, are relevant steps. Other options are graduated compression stockings (GCS) and early thrombus removal [1].

4.1 Graduated compression stockings

GCS are designed for ambulatory patients and manufactured under strict medical and technical specifications to provide a defined level of ankle compression which decreases upwards. Graduated compression mechanically reduces oedema, accelerates venous blood flow, and improves venous pump function [34]. Although compression therapy is generally harmless, it can lead to complications such as skin sores or in case of severe peripheral arterial disease arterial flow may be hampered. A Cochrane systematic review published in 2017, identified 10 RCTs with a total of 2361 participants that evaluated compression therapy, with an overall low methodological quality (e-table 2) [34]. Only five studies were included in a meta-analysis owing to differences in intervention types and lack of data. Three studies compared GCS (pressure of 30 to 40 mmHg at the ankle) versus no intervention. Two studies compared GCS (pressure 20 to 40 mmHg) versus placebo stockings. The use of GCS led to a clinically significant, although non statistically significant, reduction in the incidence of PTS with no reduction in the incidence of severe PTS no clear difference in DVT recurrence or PE (e-table 2). Included studies have reported no serious adverse effects and generally high compliance [34]. One study reported that thigh-length stockings did not provide protection against development of PTS better than knee-length stockings (RR 0.92, 95% CI 0.66 to 1.28; P = 0.6; 267 participants). Another trial reported that wearing GCS for two years seemed to be superior to wearing them for one year in terms of PTS incidence. The IDEAL DVT study showed that it was safe to shorten the duration of elastic compression therapy on an individualised basis after deep vein thrombosis for prevention of PTS, this trial however did not include an untreated control group and showed that individualized duration of compression stocking use, limited to one year in selected subjects, was as effective as 2.5 years of persistent use afteracute

DVT. The main outcome of this study was the cost-effectiveness of this strategy [35]. A pre-specified sub-study also showed that immediate compression therapy after DVT with either multilayer bandaging or compression hosiery (pressure, 35 mm Hg) was associated with a 20% absolute reduction of RVO and an 8% absolute reduction of PTS at 24 months, a relevant outcome with a non-invasive and simple measure [36].

4.2 Early thrombus removal

Anticoagulants do not remove the thrombus but only limit its extension allowing the fibrinolytic system to dissolve the thrombus fibrin mesh. Since the 1990s, an alternative approach of dissolving or removing the thrombus immediately has been proposed with the aim of preventing venous valvular damage and vein scarring, thus potentially preventing PTS development [37].

Systemic thrombolysis was the first approach. A Cochrane systematic review showed that complete clot lysis occurred significantly more often in the thrombolysis group when compared to anticoagulation alone, with considerably less PTS, with less ulceration, although the data were limited by small numbers [38]. However, systemic thrombolysis was associated with significantly more bleeding complication. There was no significant effect on mortality detected at either early or intermediate follow-up [38] (e-table 2). Systemic thrombolysis is now not commonly used, and catheter-directed thrombolysis (CDT) is the preferred treatment method. CDT as an alternative/adjunct treatment has been used since the ‘90s. Various CDT techniques are now available such as continuous infusion, ultrasound accelerated, and pulse-spray infusion of fibrinolytic drugs (streptokinase, urokinase, recombinant tissue type -Plasminogen Activator -rt-PA) delivered directly into, or near to, the thrombus to enhance thrombolysis with reduced doses, with reduced bleeding risks. The use of rt-PA seems more thrombus specific as rt-PA can convert plasminogen into plasmin efficiently only on the fibrin surface, with plasmin generated at the fibrin surface partially protected from inactivation by circulating alfa-2-antiplasmin [38]. CaVenT was the first randomized trial to assess the clinically relevant efficacy of additional catheter-directed

thrombolysis (CDT) in patients with iliofemoral DVT. Additional thrombolytic treatment reduced PTS compared with anticoagulation alone during a follow-up of 24 months, but was associated with a small additional risk of bleeding. However because of small sample size (only 209 patients), loss to follow-up and handling of missing data, the final study population was closer to the critical limit for detection of a clinical effect, and the effect estimate was imprecise [39]. Another approach for early thrombus removal is pharmacomechanical thrombectomy (PMT). A fibrinolytic drug or an anticoagulant is delivered directly into the thrombus by a catheter with concomitant thrombus aspiration or maceration. The latter can be performed by various devices such as rotating motorized systems and rheolytic instruments. Adjunctive therapy can be performed in persistent obstructive lesions such as endovenous procedures, balloon dilatation, and stenting. PMT has many theoretical advantages over CDT, in particular, shorter treatment times with shorter hospital stay, a lower dosage of the thrombolytic drug with lower systemic side effects, complete thrombus resolution, incremental cost effectiveness ratio, fewer venographic examinations [40].

The largest RCT on PMT was The National Institutes of Health (NIH)-sponsored, phase III, multi-centre (56 in USA) ATTRACT (Acute venous thrombosis: thrombus removal with adjunctive catheter-directed thrombolysis) to determine whether the use of pharmacomechanical catheter-directed thrombolysis (PCDT) added to standard care for above-the-knee proximal deep vein thrombosis prevents the PTS from developing over two years [41]. Rt-PA was used at a dose of <35 mg with either AngioJet Rheolytic Thrombectomy System (Boston Scientific, Minneapolis, Minn, USA) or Trellis Peripheral Infusion System (Discontinued by Covidien/ Medtronic, Minneapolis, Minn, USA) plus rt-PA for no longer than 24 h. In case of residual thrombus, balloon maceration, catheter aspiration thrombectomy, percutaneous transluminal balloon venoplasty, stent placement (iliac or common femoral vein), or a combination of procedures to clear residual thrombus and treat obstructive lesions were performed.

Stenting was performed if > 50% narrowing of vein diameter, robust collateral filling, or a mean

pressure gradient of more than 2 mm Hg was noted during venography. The trial data are shown in e-table 2 : no difference in PTS rate was detected between the two arms at two years (p=0.56) with more instances of bleeding (4.5%) in the interventional arm vs. 1.7% in the control arm (p=0.049) with no fatal or intracranial bleeds in either arm. PCDT was also less effective in patients older than 65 years (p=0.038). A post-hoc analysis showed that in patients with acute iliac-femoral DVT, PCDT did not influence the occurrence of PTS or recurrent venous thromboembolism, but significantly reduced early leg symptoms with lower proportion of patients who developed moderate-or-severe PTS and resulted in more significant improvement in venous disease-specific quality over 24 months [40]. More recently, the multicentre, randomised, single-blind, allocation-concealed, parallel-group, superiority CAVA trial in 15 hospitals in the Netherlands randomised 184 participants with iliofemoral DVT to ultrasound-accelerated CDT or standard care alone [42]. One-year rates of PTS were 29% and 35% in the intervention and standard care groups, respectively, a non-significant difference. In this trial, there was a long-time-interval between symptom-onset and treatment, and it did not differentiate between descending and ascending iliac-femoral DVT. Moreover, the sample size was relatively small, and follow-up was only one year; thus, CAVA was underpowered to detect a meaningful difference.

5. Treatment of PTS

5.1. Conservative treatment

First-line treatment should be conservative therapy [4].

5.1.1. Compression stockings

Compression therapy is the mainstay of treatment for venous ulcers, which may be a complication of PTS. A Cochrane systematic review identified four trials, with 116 participants, investigating the effectiveness of compressing therapies for PTS, all of low methodological quality [43] (e-table 2) . Two trials studied the effect of GCS with one study showed an improvement of PTS symptoms and

one showed no benefit. Two other trials studied the effect of an intermittent pneumatic compression device. Both reported an improvement in PTS severity [43].

A trial of an intermittent compression device may be reasonable for patients with moderate or severe PTS and edema [37], although the evidence for the efficacy of this approach is very limited.

5.1.2. Pharmacological therapy: Veno-active drugs and anticoagulation

Only four randomized trials have been performed to evaluate the effectiveness of pharmacological therapy for PTS : only 3 parallel trials and one crossover study . These studies presented a high degree of inconsistency [44] (e-table 2). Overall, medical therapy with veno-active drugs (rutosides, hidrosmin, and defibrotide) is not recommended because of the lack of evidence of positive effects and the possibility of long-term side effects.

The anticoagulant strategies have not been evaluated for the treatment of patients who have established PTS. However, it plays a crucial role in both primary (class I, level of evidence C) and secondary PTS prevention (class I, level of evidence B) [37].

5.1.3. Exercise training

The goal of exercise training is to improve symptoms by improving the calf muscle pump function and ejection of venous blood from the limb. Two studies have investigated the efficacy of exercise in patients with PTS compared with a control group that did not exercise. The methodological quality of these studies was very low) with very limited sample size (e-table 2). The exercise group showed improved symptoms and quality of life, leg strength, and mobility [45,46]. It is also essential to note that exercise does not appear to aggravate leg symptoms after DVT or increase the risk of PTS [47].

5.2. Endovascular and surgical treatment

5.2.1. Endovascular treatment of obstruction/occlusion

Multiple societies like the American Heart Association [37], European Society of Vascular Surgery [47] and the German Angiology Society [48], and American Venous Forum [49] recommend

venous angioplasty and stent recanalization in addition to standard compression therapy to aid in venous ulcer healing and to prevent recurrence in a patient with inferior vena cava or iliac vein chronic total occlusion or stenosis > 50%, with or without lower extremity deep venous reflux disease, that is associated with skin changes at risk for venous leg ulcer (C4b), healed venous leg ulcer (C5), or active venous leg ulcer (C6) (Level – 1 recommendation and evidence C) [48,49].
The Cardiovascular and Interventional Radiological Society of Europe (CIRSE) Standards of Practice Guidelines on Iliocaval Stenting recommend endovascular treatment in patients with CEAP clinical class 3 if compression therapy has failed [50].
However, the AHA recommendation on endovenous stenting for the treatment of severe chronic venous obstruction (CVO) is weak (class IIb, level of evidence B) [40]. The AHA guideline recommends stenting for severe PTS. Venous stents compared to arterial ones are characterized by high flexibility and strong radial force [51]. So far, there have been many observational studies on the treatment of CVO with dedicated venous stents , most with limited samples size (20-100 patients) as well a meta-analysis [51], creating evidence for the safety and efficacy of the revascularization treatment. At the same time, no randomized clinical trials are available yet comparing either different stents or stents with conservative treatment.
More recently, a systematic review and meta-analysis summarised the results of observational studies of efficacy and safety of venous stents in PTS with obstruction in iliofemoral venous segments [52]. Overall, 504 limbs of 489 patients from seven observational studies were included in this study. A GRADE assessment showed the quality of the evidence was "very low" for 11 relevant outcomes (e-table 2) [52].
The technical success rate was 95% but the rate of complications including 30 day thrombotic event, per-operative venous injury, and back pain was 3.4%, 18.14%, and 52%, respectively. The rates of ulcer healing, pain and oedema relief were 75.66%, 52%, and 42%, respectively.

There are strong recommendations from the vascular societies that only approved dedicated vein stents should be used for venous recanalization because these fulfill the functional requirements.

A subgroup analysis of 136 consecutive patients with PTS from the prospective Swiss Venous Stent registry [53] evaluated the incidence of stent thrombosis , estimated from duplex ultrasound or venography reported for the time on and off anticoagulation. Median follow-up was 20 (interquartile range [IQR] 9-40) months. Anticoagulation was stopped in 43 (32%) patients after 12 (IQR 6-14) months. Cumulative incidence of stent thrombosis was 13.7% (95% confidence interval [CI] 7.8-19.6%) and 21.2% (95% CI 13.2-29.2%) during the first 6 and 36 months, respectively. Age < 40 years (HR 2.26, 95% CI1.03-4.94), stents below the common femoral vein (HR 3.03, 95% CI 1.28-7.19), and PTS inflow veins (HR 2.92, 95% CI 1.36-6.25) were associated with increased incidence of stent thrombosis.

5.2.2. Open surgical reconstruction and hybrid operations

Only small, nonrandomized studies with low case numbers are available for these procedures, leading to a weak guideline recommendation (class IIb, level of evidence C) [37]. Hybrid procedures (operative endophlebectomy in addition to venous recanalization) might be required when PTS trabeculations are detected in the common femoral vein that covers the ostium of the deep femoral vein. These procedures provide an adequate inflow into the recanalized venous tract and sufficient drainage of blood from the peripheral venous system. Hybrid procedures are burdened with a significantly higher risk for complications.

In the study of van Vuuren et al. [54], which involved 109 legs treated by hybrid surgery, after 36 months, the primary, assisted primary, and secondary patency rates were observed 37%, 62%, and 72%, respectively. There was a high incidence of wound-related complications (wound infection - 27%; lymphorrhea - 33%, and wound dehiscence – 12%)

REFERENCES

1) Kahn SR, Partsch H, Vedantham S, Prandoni P, Kearon C. Subcommittee on Control of Anticoagulation of the Scientific and Standardization Committee of the International Society on Thrombosis and Haemostasis. Definition of post-thrombotic syndrome of the leg for use in clinical investigations: a recommendation for standardization. *J Thromb Haemost*. 2009;7(5):879-883.

2) Villalta S, Bagatella P, Piccioli A, Lensing AWA, Prins MH, Prandoni P. Assessment of validity and reproducibility of a clinical scale for the post-thrombotic syndrome. *Haemostasis*.1994; 24(1Suppl):158a

3) Kahn SR. Measurement properties of the Villalta scale to define and classify the severity of the postthrombotic syndrome. *J Thromb Haemost*. 2009;7(5):884-888.

4) Kahn SR. How I treat postthrombotic syndrome. *Blood*. 2009;114(21):4624-4631.

5) Jünger M, Steins A, Hahn M, Häfner HM. Microcirculatory dysfunction in chronic venous insufficiency (CVI). *Microcirculation*. 2000;7(6 Pt 2):S3-S12.

6) Steins A, Hahn M, Jünger M. Venous leg ulcers and microcirculation. *Clin Hemorheol Microcirc.*2001; 24(3):147-153

7) Coleridge Smith PD. Deleterious effects of white cells in the course of skin damage in CVI. *Int Angiol.* 2002;21(2 Suppl 1):26-32

8) Franzeck UK, Haselbach P, Speiser D, Bollinger A. Microangiopathy of cutaneous blood and lymphatic capillaries in chronic venous insufficiency (CVI). *Yale J Biol Med*. 1993;66(1):37-46

9) Pocock ES, Alsaigh T, Mazor R, Schmid-Schönbein GW. Cellular and molecular basis of Venous
insufficiency. *Vasc Cell*. 2014;6(1):24.

10) Busuttil A, Lim CS, Davies AH. Post Thrombotic Syndrome. *AdvExp Med Biol* - Advances in Internal Medicine 2016; DOI 10.1007/5584_2016_126

11) Rabinovich A, Kahn SR. The postthrombotic syndrome: current evidence and future challenges. *J Thromb Haemost*. 2017;15(2):230-241.

12) Sartori M, Favaretto E, Cini M, Legnani C, Palareti G, Cosmi B. D-dimer, FVIII and thrombotic burden in the acute phase of deep vein thrombosis in relation to the risk of post-thrombotic syndrome. *Thromb Res.* 2014;134(2):320-5

13) Prandoni P, Lensing AWA, Prins MH, Pesavento R, Piccioli A, Sartori MT et al. The impact of residual thrombosis on the long-term outcome of patients with deep venous thrombosis treated with conventional anticoagulation. *Semin Thromb Hemost*. 2015;41(2):133-140.

14) Yamaki T, Nozaki M, Sakurai H, Takeuchi M, Soejima K, Kono T. High peak reflux velocity in the proximal deep veins is a strong predictor of advanced post-thrombotic sequelae. *J Thromb Haemost*. 2007;5(2):305-312.

15) Haenen JH, Janssen MC, Wollersheim H, Van't Hof MA, de Rooij MJ, van Langen H et al. The development of postthrombotic syndrome in relationship to venous reflux and calf muscle pump dysfunction at 2 years after the onset of deep venous thrombosis. *J Vasc Surg*. 2002;35(6):1184-1189.

16) Jeraj L, Ježovnik MK, Poredoš P. Insufficient Recanalization of Thrombotic Venous Occlusion-Risk for Postthrombotic Syndrome. *J VascIntervRadiol.* 2017;28(7):941-944.

17) Ježovnik MK, Poredoš P. Factors influencing the recanalisation rate of deep venous thrombosis *.Int Angiol*. 2012; 31 (2):169-175.

18) Rabinovich A, Cohen JM, Kahn SR. Predictive value of markers of inflammation in the postthrombotic syndrome: a systematic review: inflammatory biomarkers and PTS. *Thromb Res*. 2015;136(2):289-297.

19) Chitsike RS, Rodger MA, Kovacs MJ, Betancourt MT, Wells PS, Anderson DR, et al. Risk of post-thrombotic syndrome after subtherapeutic warfarin anticoagulation for a first unprovoked deep vein thrombosis: results from the REVERSE study. *J Thromb Haemost*. 2012;10(10):2039-2044.

20) van Dongen CJ, Prandoni P, Frulla M, Marchiori A, Prins MH, Hutten BA. Relation between quality of anticoagulant treatment and the development of the postthrombotic syndrome. *J Thromb Haemost*. 2005;3(5):939-942.

21) Kahn SR, Shrier I, Julian JA, Ducruet T, Arsenault L, Miron MJ, et al. Determinants and time course of the postthrombotic syndrome after acute deep venous thrombosis. *Ann Intern Med*. 2008;149(10):698-707.

22) Baglin T. Prevention of post-thrombotic syndrome: a case for new oral anticoagulant drugs or for heparins?*J ThrombHaemost*. 2012;10(8):1702-1703.

23) Hull RD, Pineo GF, Brant R, Liang J, Cook R, Solymoss S et al. Home therapy of venous thrombosis with long-term LMWH versus usual care: patient satisfaction and post-thrombotic syndrome. *Am J Med*. 2009;122(8):762-769.e3.

24) Hull RD, Liang J, Townshend G. Long-term low-molecular-weight heparin and the post-thrombotic syndrome: a systematic review. *Am J Med*. 2011;124(8):756-765.

25) Jeraj L, Jezovnik MK, Poredos P. Rivaroxaban versus warfarin in the prevention of post-thrombotic syndrome. *Thromb Res*. 2017;157:46-48.

26) Cheung YW, Middeldorp S, Prins MH, Pap AF, Lensing AW, Ten Cate-Hoek AJ, et al. Post-thrombotic syndrome in patients treated with rivaroxaban or enoxaparin/vitamin K antagonists for acute deep-vein thrombosis. A post-hoc analysis. *Thromb Haemost*. 2016;116(4):733-738.

27) Søgaard M, Nielsen PB, Skjøth F, Kjældgaard JN, Coleman CI, Larsen TB. Rivaroxaban Versus
Warfarin and Risk of Post-Thrombotic Syndrome Among Patients with Venous Thromboembolism. *Am J Med*. 2018;131(7):787-794.e4.

28) Prandoni P, Ageno W, Ciammaichella M, Mumoli N; Zanatta N, Imberti D et al. The risk of post-thrombotic syndrome in patients with proximal deep vein thrombosis treated with the direct oral anticoagulants. *Intern Emerg Med*. 2020;15(3):447-452.

29) Palareti G, Cosmi B. The direct oral anticoagulants may also be effective against the risk of postthrombotic syndrome. *Intern Emerg Med*. 2020;15(3):365-367.

30) Heit JA. Venous thromboembolism: disease burden, outcomes and risk factors. *J Thromb Haemost*. 2005;3(8):1611-1617.

31) Silverstein MD, Heit JA, Mohr DN, Petterson TM, O'Fallon WM, Melton LJ 3rd. Trends in the incidence of deep vein thrombosis and pulmonary embolism: a 25-year population-based study. *Arch Intern Med*. 1998;158(6):585-593.

32) Cohen AT, Agnelli G, Anderson FA, Arcelus JI, Bergqvist D, Brecht JG, et al. Venous thromboembolism (VTE) in Europe. The number of VTE events and associated morbidity and mortality. *Thromb Haemost*. 2007;98(4):756-764.

33) Lal BK. Venous ulcers of the lower extremity: Definition, epidemiology, and economic and social burdens. *SeminVasc Surg*. 2015;28(1):3-5.

34) Appelen D, van Loo E, Prins MH, Neumann MH, Kolbach DN. Compression therapy for prevention of post-thrombotic syndrome. *Cochrane Database Syst Rev*. 2017;9 (9):CD004174. Published 2017 Sep 26.

35) Ten Cate-Hoek AJ, Amin EE, Bouman AC, Meijer K, Tick LW, Middeldorp S, et al. Individualized versus standard duration of elastic compression therapy for prevention of post-thrombotic syndrome (IDEAL DVT): A multicenter, randomized, single-blind, allocation-concealed, non-inferiority trial. *Lancet Haematol* 2018;5 (1):e25- e33

36) Amin EE, Bistervels IM, Meijer K, Tick LW, Middeldorp S, Mostard G, et al. Reduced incidence of vein occlusion and postthrombotic syndrome after immediate compression for deep vein thrombosis. *Blood*. 2018;132(21):2298-2304.

37) Kahn SR, Comerota AJ, Cushman M, Evans NS, Ginsberg JS, Goldenberg NA, et al. The postthrombotic syndrome: evidence-based prevention, diagnosis, and treatment strategies: a

scientific statement from the American Heart Association [published correction appears in Circulation. 2015 Feb 24;131(8):e359]. *Circulation*. 2014;130(18):1636-1661.

38) Watson L, Broderick C, Armon MP. Thrombolysis for acute deep vein thrombosis. *Cochrane Database Syst Rev*. 2014;(1):CD002783. Published 2014 Jan 23.

39) Haig Y, Enden T, Grøtta O, Kløw NE, Slagsvold CE, Ghanima W, et al. CaVenT Study Group. Post-thrombotic syndrome after catheter-directed thrombolysis for deep vein thrombosis (CaVenT): 5-year follow-up results of an open-label, randomised controlled trial. Lancet Haematol. 2016 Feb;3(2):e64-71. doi: 10.1016/S2352-3026(15)00248-3. Epub 2016 Jan 6. PMID: 26853645.

40) Comerota AJ, Kearon C, Gu C, Julian JA, Goldhaber SZ, Kahn SR, et al. Endovascular Thrombus Removal for Acute Iliofemoral Deep Vein Thrombosis. *Circulation*. 2019;139(9):1162-1173.

41) Comerota AJ, Kearon C, Gu C, Julian JA, Goldhaber SZ, Kahn SR, et al. Pharmacomechanical Catheter-Directed Thrombolysis for Deep-Vein Thrombosis. *N Engl J Med*. 2017;377(23):2240-2252.

42) Notten P, Ten Cate-Hoek AJ, Arnoldussen CWKP, Strijkers RHW, de Smet AAEA, Tick LW, et al. Ultrasound-accelerated catheter-directed thrombolysis versus anticoagulation for the prevention of post-thrombotic syndrome (CAVA): a single blind, multicentre, randomised trial. *Lancet Haematol*. 2020;7(1):e40-e49.

43) Azirar S, Appelen D, Prins MH, Neumann MH, de Feiter AN, Kolbach DN. Compression therapy for treating post-thrombotic syndrome. *Cochrane Database Syst Rev*. 2019;9(9):CD004177. Published 2019 Sep 18.

44) Morling JR, Broderick C, Yeoh SE, Kolbach DN. Rutosides for treatment of post-thrombotic syndrome. *Cochrane Database Syst Rev*. 2018;11(11):CD005625. Published 2018 Nov 8.

45) Padberg FT Jr, Johnston MV, Sisto SA. Structured exercise improves calf muscle pump function in chronic venous insufficiency: a randomized trial. *J Vasc Surg*. 2004;39(1):79-87.

46) Kahn SR, Shrier I, Shapiro S, Houweling AH, Hirsch AM, Reid RD et al. Six-month exercise training program to treat post-thrombotic syndrome: a randomized controlled two-centre trial. *CMAJ*. 2011;183(1):37-44. 46) Shrier I, Kahn SR, Steele RJ. Effect of early physical activity on long-term outcome after venous thrombosis. *Clin J Sport Med*. 2009;19(6):487-493.

47) Wittens C, Davies AH, Bækgaard N, et al. Editor's Choice - Management of Chronic Venous Disease: Clinical Practice Guidelines of the European Society for Vascular Surgery (ESVS) [published correction appears in Eur J VascEndovasc Surg. 2020 Mar;59(3):495]. *Eur J Vasc Endovasc Surg*. 2015;49(6):678-737.

48) Lichtenberg M, de Graaf R, Erbel C. Standards for recanalisation of chronic venous outflow obstructions. *Vasa*. 2018;47(4):259-266.

49) O'Donnell TF Jr, Passman MA, Marston WA, Ennis WJ, Dalsing M, Kistner RL et al. Management of venous leg ulcers: clinical practice guidelines of the Society for Vascular Surgery ® and the American Venous Forum. *J Vasc Surg*. 2014;60(2 Suppl):3S-59S.

50) Mahnken AH, Thomson K, de Haan M, O'Sullivan GJ. CIRSE standards of practice guidelines on iliocaval stenting. *Cardiovasc Intervent Radiol*. 2014;37(4):889-897.

51) Razavi MK, Jaff MR, Miller LE. Safety and Effectiveness of Stent Placement for Iliofemoral Venous Outflow Obstruction: Systematic Review and Meta-Analysis. *Circ Cardiovasc Interv*. 2015;8(10):e002772.

52) Qiu P, Zha b , Xu A, Wang W , Zhan Y, Zhu X et al. Systematic Review and Meta-Analysis of Iliofemoral Stenting for Postthrombotic Syndrome. *Eur J Vasc Endovasc Surg*. 2019;57(3):407-416.

53) Sebastian T, Spirk D, Engelberger RP, Dopheide JF, Baumann FA, Barco S, et al. Incidence of Stent 3131 Thrombosis after Endovascular Treatment of Iliofemoral or Caval Veins in Patients with the Postthrombotic Syndrome.

Thromb Haemost. 2019;119(12):2064-2073.

54) van Vuuren T, de Wolf MA, Arnoldussen C, Kurstjens RL, van Laanen JH, Jalaie H et al. Editor's Choice - Reconstruction of the femoro-ilio-caval outflow by percutaneous and hybrid interventions in symptomatic deep venous obstruction. *Eur J Vasc Endovasc Surg*. 2017;54(4):495-503.31
