# Supplementary material for: The Post-thrombotic Syndrome-Prevention and Treatment: VAS-European Independent Foundation in Angiology/Vascular Medicine Position Paper
Source: Front Cardiovasc Med. 2022 Feb 24;9:762443. doi: 10.3389/fcvm.2022.762443 (PMC8907532; doi:10.3389/fcvm.2022.762443)
Supplement: Supplementary file 2 [file Table_1.DOCX]

**E-Table 1:Characteristics of studies on PTS diagnosis and surveillance**

| **Source** | **Inclusion criteria** | 1. **PTS Elements and scoring** 2. **Timing of PTS assessment** | Sample size | Interater reliability | **External validation** | **Rational clinical examination level of evidence** |
| --- | --- | --- | --- | --- | --- | --- |
| Villalta et al. 1994 | First episode of proximal DVT | 1) -*symptoms assessment by patient self-report*:  pain, cramps, heaviness, pruritus , paresthesia;  -*signs by clinician assessment*:edema, skin induration, hyperpigmentation, venous ectasia, redness, pain during calf compression,  -Severity of each symptom and sign rated as:  0 (absent), 1 (mild), 2 (moderate), or 3(severe)  0–4 No PTS; 5–9 Mild PTS; 10–14 Moderate PTS  15 or more, or presence of ulcer: severe PTS.  In some studies two consecutive assessments that corresponded to PTS (score >5) were required to diagnose PTS , in other studies PTS has been diagnosed based on a single assessment.  2) 6-36 mo. | 100 | 3 studies:  K: 0.78-1.00 | Yes | 5 |
| Brandjes et al. 1997 | First episode of proximal DVT | 1. *Subjective criteria*   -Mild to moderate ( > 3) (including one objective criterion)  Spontaneous pain in the calf Spontaneous pain in the thigh Pain in the calf on standing/walking Pain in the thigh on standing/walking Oedema on the foot/calf Heaviness of the leg -Severe PTS ( score > 4)  Spontaneous pain and pain on standing/walking  Oedema of calf Impairment of daily activities  *-Objective criteria*  Calf circumference increased by 1 cm  Ankle circumference increased by 1 cm; Pigmentation ; Venectasia; Newly formed varicosis; Phlebitis; Pigmentation, discolouration and venectasia  2) every 3 mo. at the outpatient clinic during the first 2 yrs, and every 6 months thereafter for at least 5 yrs. | 194 | Not determined | Not determined | 5 |
| Ginsberg et al. 2001 | First episode of proximal DVT | 1) chronic (>1 month in duration),  typical (better after a night's sleep and leg elevation, worse at the end of the day and after prolonged standing or sitting), and pain and swelling of the leg(s) 6 months or more after a proximal DVT.  Patients were categorized as having PTS only if they had both pain and swelling.  3) every 3-6 months | 202 | Not determined | Not determined | 5 |
