# Supplementary material for: The Post-thrombotic Syndrome-Prevention and Treatment: VAS-European Independent Foundation in Angiology/Vascular Medicine Position Paper
Source: Front Cardiovasc Med. 2022 Feb 24;9:762443. doi: 10.3389/fcvm.2022.762443 (PMC8907532; doi:10.3389/fcvm.2022.762443)
Supplement: Supplementary file 3 [file Table_2.DOCX]

**e-table 2**

**GRADE evidence profile of systematic reviews and RCTs for PTS prevention and treatment**

|  |  | Quality assessment | | | | | Summary of findings | | | |
| --- | --- | --- | --- | --- | --- | --- | --- | --- | --- | --- |
| **Systematic reviews** | Limitations | Inconsistency | Indirectness | Imprecision | Publication bias | Overall rating | N of pts | N of pts | RR | Absolute risk |
| **GCS vs control for PTS prevention**(Placebo /no GCS) |  |  |  |  |  |  | **GCS** | **Control** |  |  |
| ***Appelen , 2017***  **Cumulative incidence of any PTS**  (follow-up 2-6.3 y)  (5RCTs of 10) | No  serious | No  serious | No  serious | No  serious | undetected | low | 703 | 690 | **0.62**  95% CI:  0.38-1.01; P=0.05 | **400 per 1000 with control**  **vs 248 per 1000 with GCS**  (152 to 404) |
| ***Appelen , 2017***  **Cumulative incidence of severe PTS**  (follow-up 2-6.3 y)  (4RCTs of 10) | No  serious | No  serious | No  serious | No  serious | undetected | low | 409 | 394 | 0.78,  (95% CI: 0.53 -1.15) | **86 per 1000 with control**  **vs**  **67 per 1000** (46-99) with GCS |
| **Systemic thrombolysis (adjunctive)vs standard anticoagulation alone for PTS prevention** |  |  |  |  |  |  | **Thrombolysis** | **Standard**  **anticoagulation** |  |  |
| ***Watson et al. 2014***  **Incidence of**  PTS (17 RCTS)  Leg ulcers (3 RCTs)  Bleeding (15 RCTS) | No  serious | Moderate | No  serious | No  serious | undetected | low | 87  104  454 | 65  84  325 | 0.55; 95% CI: 0.41-0.73; P< 0.0001  0.48; 95% CI: 0.12-1.88  2.18; 95% CI 1.37-3.47, P= 0.001 | --  --  -- |
| **Catheter -directed thrombolysis (adjunctive) vs standard anticoagulation alone for PTS prevention**  ***Watson et al 2014***  **incidence of**  PTS (1 RCTs)  Leg ulcers (1 RCTs)  Bleeding (2 RCTS) |  |  |  |  |  |  | 90  90  108 | 99  99  116 | 0.74, 95% CI: 0.55-1.00  not estimable  7.69 ; 95 CI: 0.40-1.47 | -  -- |
| **Compression therapy for PTS treatment** |  |  |  |  |  |  | **Compression** | **No compression** |  |  |
| ***Azirar et al 2019***  **Severity of PTD**  (follow-up: 0–25.6 mo)  Compression therapy vs. placebo stockings or no compression  2 RCTS | No serious | No serious | No serious | No serious | undetected | Very low | 69 | 2 small studies of short duration: 1 reported benefit and 1 no benefit from GCS use | --- | --- |
| ***Azirar et al 2019***  Medical compression device vs.control device  **Severity of PTS**  (follow-up: 8–20 we)  (2 RCTs) |  |  |  |  |  |  | 47 | Control devices used on the same participants that used medical  device | --- | --- |
| **Rutosides vs placebo or not treatment for PTS treatment**  ***Morling et al 2018***  **Improvement in PTS**  (8 we - 12 mo) 2 RCT | No serious | Moderate | No serious | No serious | undetected | Very low | 164 |  | 1.29;95% CI 0.69 - 2.41 | 554 per 1000 vs 616 per 1000  (462 to 750) |
| **Ilio-femoral stenting for PTS treatment** |  |  |  |  |  |  | --- | **Stents** |  |  |
| ***Qiu et al, 2019;***  7 observational studies  Ulcer healing  Pain relief  Oedema relief  Peri-operative venous injury  30-day thrombotic events  Technical success | Serious | Serious | Serious | Serious | undetected | Very low | 76%; 95%CI:67-83%  52%; 95%CI:32-72%  42%; 95%CI:33-52%  18%; 95% : 8-36%  3.4%; 95%CI:1.6-7%  95%; 95%CI:91-98% | 124 limbs  81 limbs  99 limbs  304 limbs  251 limbs  413 limbs | --- | --- |
| **Randomized clinical trials**  **PTS prevention** | **Random**  **sequence**  **generation** | **Allocation**  **concealment** | **Blinding of**  **participants and personnel** | **Blinding of outcome assessment** | **Incomplete**  **outcome**  **data** | **Selective reporting** | **Risk of bias** | **PTS**  **diagnosis** | N .pts | RR |
| **ATTRACT**  **Any PTS at 6-24 mo**  (PMT Vs. standard anticoagulation)  Major bleeding | central computer generation | yes | no | yes | 5/337 vs 11/355 | low | low | VS | 157/336 (47%)  17/355 (48%)  6 (1.7%) vs 1 (0.3%9 | 0.96 (95% CI :0.82-1.11)  6.18 (95% CI:0.78-49.2)  p =0.049 |
| **CAVA**  **Any PTS at 12 mo**  (additional thrombolysis  Vs. standard anticoagulation)  Major bleeding | web based | yes | no | Not specified | 58/77 vs 57/75 | moderate risk | moderate | VS | 22/77 (29%) vs  26/75% (35%)  4 (5%) vs 0 | 0.75 (95%CI:0.30-5.10) |
| **PTS treatment**  **Exercise**  *Padberg et al, 2004*  Physical therapy to strengthen calf musculature | Previously prepared randomization list | No | No | Not specified | 28/31  Vs  13/13 | Moderate risk | high | Venous Clinical Severity Score | 13 vs 18 | Non change in quality of life or severity scores over 6 months  ( calf muscle function improved) |
| **PTS treatment**  **Exercise**  *Kahn et al, 2011*  6 –mo trainer supervised program vs  Education session with monthly telephone follow-up | Web base randomization lisr | yes | No | Yes | 39/43 | Moderrate risk | high | VS | 22 vs 21  ( 20 vs 15) | Exercise training mean change of VS at 6 months:  -3.6 vs -1.6 , difference -2.0  95% CI: -4.6 + 0.6, p= 0.14 |
